# Impending central retinal vein occlusion and granulomatous uveitis in a patient with Satoyoshi syndrome

**DOI:** 10.1097/MD.0000000000033284

**Published:** 2023-03-17

**Authors:** Yoshinari Saima, Yoshiaki Tanaka, Akihiro Kakehashi, Toshikatsu Kaburaki

**Affiliations:** a Department of Ophthalmology, Jichi Medical University Saitama Medical Center, Saitama, Japan; b Division of Ophthalmology, Department of Visual Sciences, Nihon University School of Medicine, Tokyo, Japan.

**Keywords:** care report, central retinal vein occlusion, granulomatous uveitis, papillitis, Satoyoshi syndrome

## Abstract

Satoyoshi syndrome is a rare systemic autoimmune disease that presents with painful muscle spasms. We report a case of impending central retinal vein occlusion (CRVO) secondary to granulomatous pan-uveitis in a 32-year-old woman with Satoyoshi syndrome.

**Patient concerns:**

At the age of 7 years, she developed generalized hair loss and painful spasms and was diagnosed with Satoyoshi syndrome. Her current symptoms included sudden metamorphopsia and decreased visual acuity in the left eye. She visited our hospital with extensive intraretinal hemorrhage (including the macula), meandering of the retinal veins, and swelling of the optic disc. Fluorescence fundus angiography demonstrated hyper fluorescence of the optic disc and leakage from the retinal veins, suggesting CRVO associated with optic papillitis and segmental periphlebitis.

**Diagnoses:**

In the left eye, there were 2 + cells in the anterior chamber and 1 + in the anterior vitreous.

**Interventions:**

We increased the existing dose of prednisolone for the treatment of uveitis and started her on oral aspirin and kallidinogenase for CRVO.

**Outcomes:**

The impending CRVO gradually subsided, and her visual acuity improved. However, during the subsequent treatment course, angle nodules were observed in the left eye, and the intraocular pressure (IOP) gradually increased. Although the angled nodules disappeared with topical corticosteroid treatment, the IOP did not reduce and became uncontrolled. Therefore, we performed trabeculotomy first, followed by trabeculectomy, after which the IOP decreased to approximately 10 mm Hg.

**Lessons:**

Unilateral granulomatous pan-uveitis and impending CRVO were observed in this patient. Several cases of Satoyoshi syndrome complicated by various autoimmune or immunological disorders have been reported. However, to the best of our knowledge, no reports of Satoyoshi syndrome presenting with uveitis or CRVO have been published. Physicians should consider uveitis as a complication of Satoyoshi syndrome.

## 1. Introduction

Satoyoshi syndrome, also known as systemic Komura-Gaeri syndrome, is a rare progressive neurological disease that presents with various complicated symptoms, including painful intermittent muscle spasms, diarrhea, hair loss, musculoskeletal abnormalities, and amenorrhea.^[1,2]^ Although this disease was initially reported in 1967, only 64 cases have been reported worldwide as of 2018, and 77 cases as of 2021.^[3,4]^ Corticosteroid therapy is effective for suppressing the muscle spasms associated with this disease to some extent; hence, autoimmune mechanisms may be associated with its pathogenesis. However, the underlying mechanisms of this disease have not yet been elucidated. ^[2–6]^ In terms of treatment, and muscle relaxants, anticonvulsants, corticosteroids, immunosuppressants, and immunoglobulin therapy have been reported to be effective in various case reports.^[3–6]^ Herein, we report a case of granulomatous pan-uveitis and impending central retinal vein occlusion (CRVO) co-occurring with Satoyoshi syndrome.

## 2. Case presentation

A 32-year-old woman with Satoyoshi syndrome presented to the ophthalmology department of our tertiary medical center after noticing sudden visual disturbance and metamorphopsia in her left eye.

At the age of age, she experienced symptoms of hair loss on her head and painful intermittent muscle spasms in her extremities. Hair loss spreads throughout the body, and the frequency of painful spasms increases. There was no diarrhea. Administration of the muscle relaxant dantrolene (10 mg/day) effectively improved muscle spasms. Peripheral blood tests revealed mild elevations in creatine phosphokinase (214 IU/L; normal range, 10–125 IU/L), antinuclear antibody (titer of > 1:1280; normal range, <1:20), and anti-DNA antibody (titer of 1:160; normal range, <1:5). Thyroid hormone, urinary cortisol, and blood testosterone levels were all within normal reference ranges, and growth hormone, follicle-stimulating hormone, luteinizing hormone, and prolactin were normally secreted based on a glucagon/thyroid-releasing hormone/luteinizing hormone-releasing hormone challenge test. Radiographs showed that the metaphyses of the long bones of the extremities were irregular. Brain and spinal cord magnetic resonance imaging demonstrated no abnormalities, and there were no abnormalities in nerve conduction velocity based on electroencephalography and electromyography findings. Electrical stimulation of the tibial and peroneal nerves caused painful muscle spasms in the extensor muscles of the ankle joint and toe, which persisted for approximately one minute after termination of the stimuli.

At the age of 7, she was diagnosed with Satoyoshi syndrome based on painful muscle spasms, systemic hair loss, and bone abnormalities. Intravenous immunoglobulin (130 mg/kg/day for 3 days) was administered every 2 weeks for a total of 3 times, following which the frequency of painful muscle spasm attacks decreased to approximately 2-3 times per month. Following the 4th course of treatment, immunoglobulin therapy was reduced to once every 2 weeks. No re-exacerbation of seizures was observed. Antinuclear antibody titers decreased to 1:160 and anti-DNA antibody titers decreased (<1:5).

When diarrhea was observed at the age of 23 years, prednisolone (5 mg/day) was initiated to treat eosinophilic gastroenteritis. The patient’s family and medical histories were unremarkable.

At the age of 32, she became aware of a sudden loss of vision and metamorphopsia in her left eye and was referred to the ophthalmology department at our tertiary medical center. At that time, she was prescribed oral prednisolone (8 mg/day), methotrexate (6 mg once per week), dantrolene (100 mg/day), and baclofen (12.5 mg/day) to address the symptoms of Satoyoshi syndrome.

Her physical findings showed generalized alopecia, bone abnormalities, and muscle spasms in the extremities, occasionally occurring once every several minutes. There were no symptoms or systemic findings, such as oral aphthae, skin symptoms, headaches, tinnitus, hearing loss, and hypertension.

**Figure 1.**
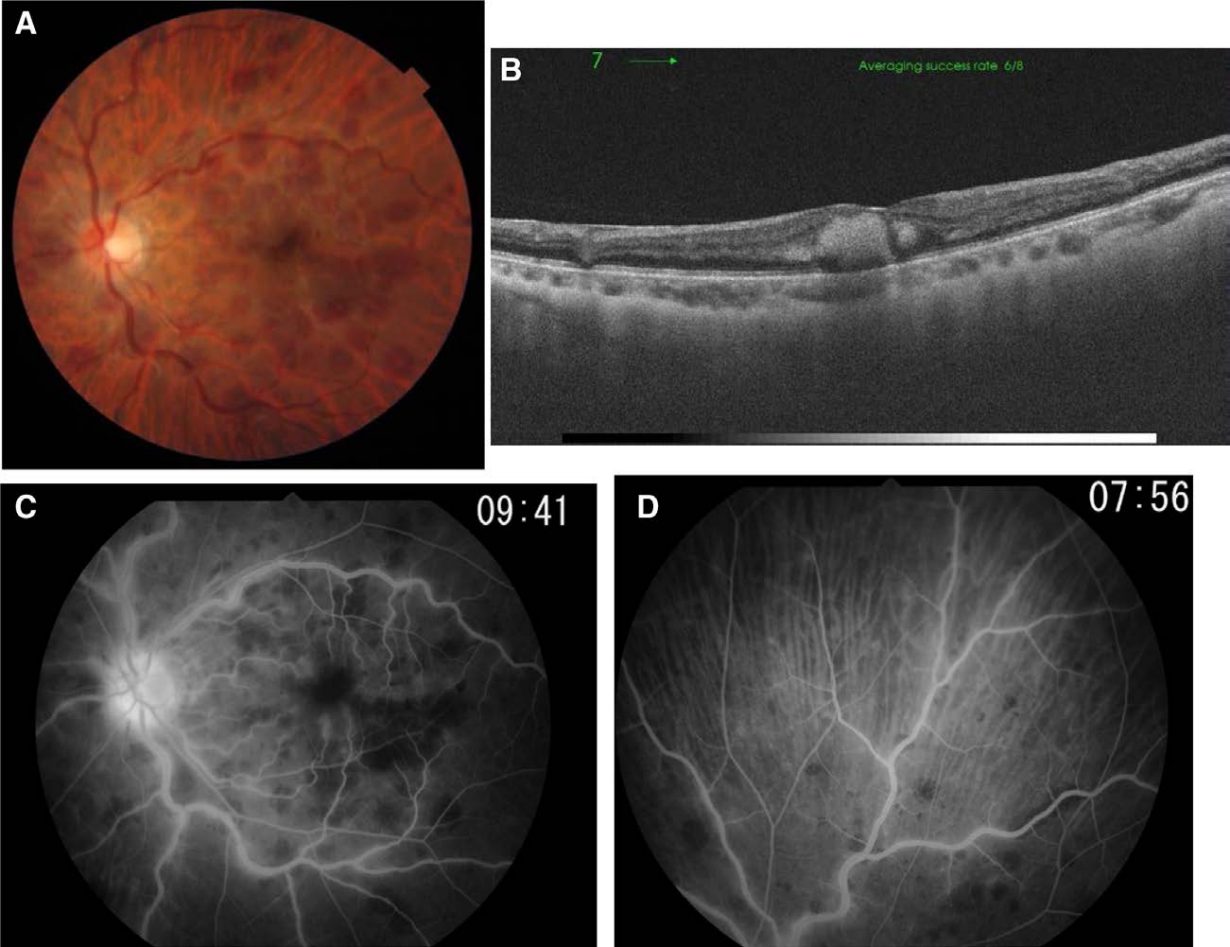
Fundus findings in the left eye at the first visit to our tertiary medical center. (A) A color fundus photograph showing many blot hemorrhages in the posterior retina as well as dilated meandering veins. (B) An optical coherence tomography image showing intraretinal hemorrhage at the fovea. (C, D) Fluorescein fundus angiography showing fluorescein leakage from the optic disc and diffuse retinal veins, and from segmental retinal veins at the site of peripheral retina.

Her corrected visual acuity at the first visit was 1.0 in the right eye and 0.07 in the left eye, respectively. Her intraocular pressure (IOP) was 13 mm Hg in the right eye and 23 mm Hg in the left eye. Two + cells in the anterior chamber and 1 + cell in the anterior vitreous were observed in the left eye. We observed extensive retinal hemorrhage (including the macula), tortuosity of the retinal arteries and veins, and swelling of the optic disc (Fig. 1A). Optical coherence tomography revealed foveal thickening due to intraretinal hemorrhage (Fig. 1B), which was thought to be the cause of vision loss and metamorphopsia. Fluorescein angiography revealed hyperfluorescence of the optic disc and leakage from the retinal veins (Fig. 1C and D), suggesting not only impending CRVO associated with papillitis but also segmental periphlebitis due to uveitis in the peripheral retina. No remarkable abnormalities were observed in her right eye.

Blood examination results revealed abnormal white blood cell counts (19,200/µL), erythrocyte sedimentation rates (44 mm/hour), and C-reactive protein levels (2.19 mg/dL). Her kidney function and liver function tests were within the normal range, as were her serum angiotensin-converting enzyme, soluble interleukin-2 receptor, creatinine, syphilis serum reaction antibody, Bartonella spp. antibody titers, anti-Toxoplasma antibody levels, and anti-HIV antibody titers. Normal chest radiographic findings, a negative purified-protein derivative reaction, and a negative T-SPOT TB test excluded the differential diagnoses of sarcoidosis and tuberculosis. Based on these results, commonly known causes of uveitis were ruled out. Therefore, Satoyoshi syndrome was tentatively speculated as a possible cause of papillitis and impending CRVO in this patient.

**Figure 2.**
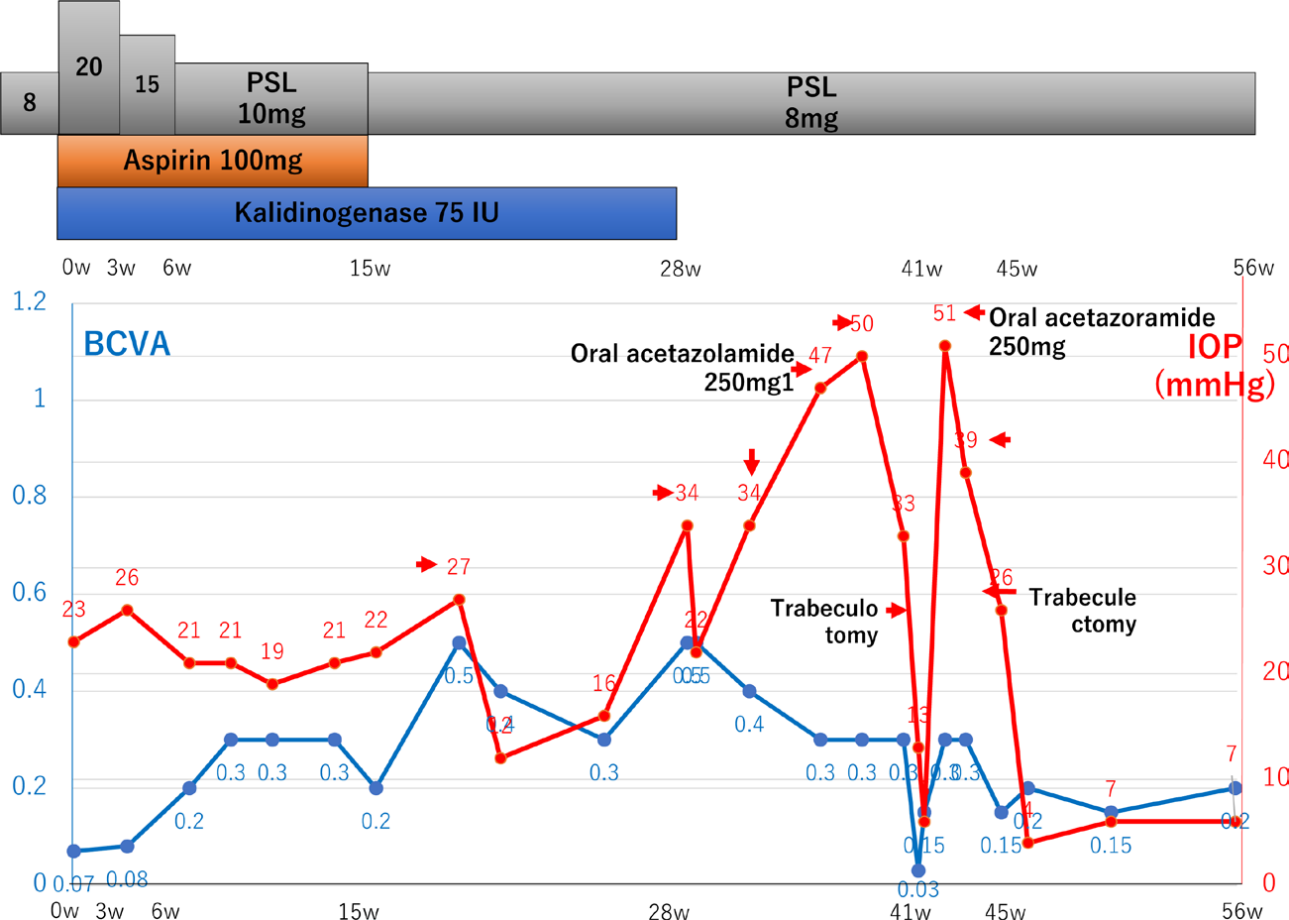
The clinical course of the left eye and the patient’s medication profile. Oral corticosteroids improved the patient’s best corrected visual acuity. However, the intraocular pressure gradually increased and became uncontrollable. Trabeculotomy and additional trabeculectomy were required.

**Figure 3.**
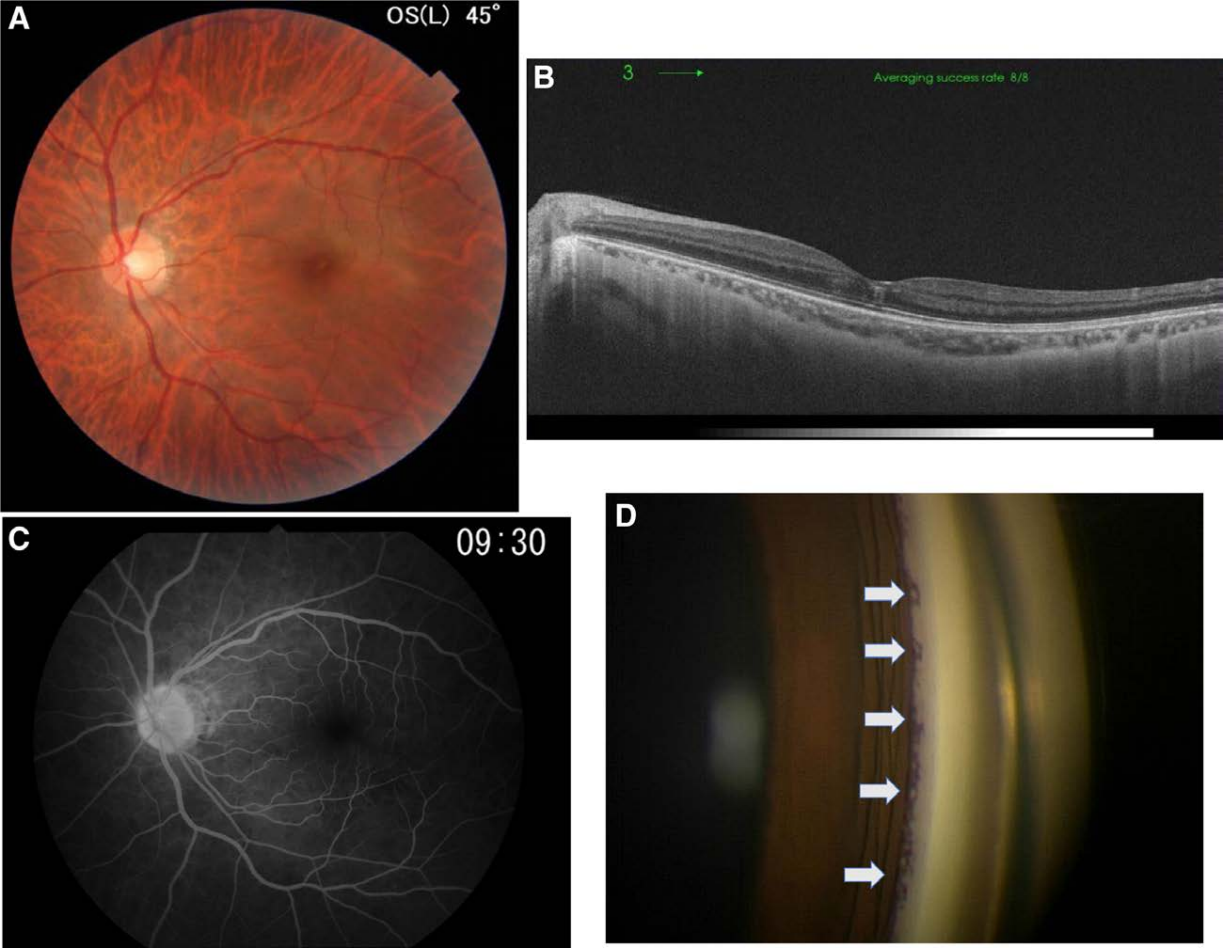
Findings for the left eye 28 weeks after the first visit. (A) A color fundus photograph showing that the blot hemorrhages in the posterior retina had disappeared. (B) An optical coherence tomography image showing that the foveal intraretinal hemorrhage had almost disappeared. (C) Fluorescein fundus angiography showing that fluorescein leakages from the optic disc and the retinal veins had disappeared. (D) Multiple angle nodules were found which led to the increase in intraocular pressure.

The prednisolone dose was increased to 20 mg/day for the treatment of uveitis, and oral aspirin (100 mg/day) and kallidinogenase (150 IU/day) for CRVO were added to her treatment regimen (Fig. 2). Tortuosity and dilation of the retinal veins improved, and the intraretinal hemorrhages at the macula and swelling of the optic disc disappeared (Fig. 3A and B). Her best corrected visual acuity improved to 0.3 in her left eye.

Fluorescein angiography revealed that prior leakages from the optic disc and retinal veins had disappeared (Fig. 3C), suggesting that the papillitis, impending CRVO, and segmental periphlebitis had improved. Twenty-eight weeks later, her visual acuity recovered to 0.5 in the left eye.

However, the IOP gradually increased to 50 mm Hg after 28 weeks, and angular nodules were observed (Fig. 3D). Although the angled nodules disappeared with topical corticosteroid treatment, IOP was not reduced. This was likely due to the increased peripheral anterior synechia. We prescribed 4 types of topical antiglaucoma eye drops along with oral acetazoramide (500 mg/day). However, because IOP was not reduced (≥40 mm Hg), we first performed trabeculotomy in the left eye 41 weeks after the start of treatment. However, because IOP was not reduced and progression of chronic angle closure was observed, trabeculectomy was performed trabeculectomy 4 weeks later, after which the IOP was reduced to approximately 10 mm Hg (Fig. 2). The patient’s final best corrected visual acuity was 0.2, with an IOP of 7 mm Hg in the left eye 56 weeks after treatment initiation.

The patient provided written informed consent for this anonymized case report. This case report was approved by the ethics committee of Jichi Medical University Saitama Medical Center.

## 3. Discussion

Satoyoshi syndrome is a rare progressive disease characterized by painful muscle spasms, diarrhea, hair loss, musculoskeletal abnormalities, stunted growth, amenorrhea, and other symptoms. It develops during childhood. A total of 77 cases have been reported worldwide thus far.^[1–4]^ Satoyoshi syndrome is considered an acquired disease as no familial onset has been reported.^[7]^ Painful muscle spasms are the most common initial symptoms.^[2,7]^ These muscle spasms are progressive and severely painful. The duration of a spasm is usually a few minutes or less; however, these spasms occur recurrently 1 to 100 times daily.^[2]^ As the disease progresses, the frequency of convulsions increases, and the site of convulsions can rise from the lower limbs to the upper limbs and finally to the head and masticatory muscles.^[2,7]^ Muscle spasms can be induced by voluntary movements such as raising the upper limbs.^[7]^ The resulting malnutrition and complications of brain or lung disease can be fatal^[2]^; for example, 10 out of 16 cases died within 10 years in an early report.^[7]^ However, in recent years, the mortality rate has improved significantly with advances in treatment.^[3,7]^

Typically, no abnormal findings are found in general blood tests, cerebrospinal fluid tests, urinalysis, or stool tests. However, antinuclear antibodies in sera are positive in some cases (especially in advanced cases).^[3]^ Moreover, abnormal electrolytes, including hypokalemia and elevated creatine kinase, may be seen.^[2]^ No obvious abnormal findings are generally found upon electroencephalography and electromyography.^[2,7]^

Muscle relaxants (dantrolene and baclofen), anticonvulsants (carbamazepine and phenytoin), systemic corticosteroids, and immunosuppressants (cyclosporine, azathioprine, methotrexate, tacrolimus, and cyclophosphamide) were administered to treat the syndrome. High-dose intravenous immunoglobulin therapy and plasma exchange are administered in severe cases.^[3–6]^

There have been case reports of Satoyoshi syndrome occurring in autoimmune and immune disorders. For example, Satoh et al^[8]^ reported a case who was complicated with systemic lupus erythematosus, myasthenia gravis, and Satoyoshi syndrome. Similarly, Asherson et al^[9]^ reported a case of eosinophilic gastroenteritis associated with Satoyoshi syndrome. There have likewise been case reports of co-occurring rheumatoid arthritis, nephritis, and bronchial asthma.^[7]^

The etiology of Satoyoshi syndrome remains unknown. This syndrome does not present with any hereditary complications and does not typically present as a complication of other immunological diseases. The effectiveness of systemic corticosteroids and immunosuppressive drugs^[3]^ as well as the typical presence of antinuclear antibodies^[10]^ and autoantibodies against brain and intestinal organs on laboratory testing^[11]^ suggests the possibility of an autoimmune origin.^[2,7]^

Because immunological disorders, such as Vogt-Koyanagi-Harada syndrome, Behçet’s disease, and systemic lupus erythematosus, are associated with uveitis, Satoyoshi syndrome may be complicated by uveitis. Wu et al^[12]^ reported a case of papillitis with diminished retinal venous outflow and intraocular inflammation that resolved with steroid therapy, and was subsequently found to be dermatomyositis. The ocular manifestations of the present case are similar to those of that case. Therefore, papillitis and impending CRVO in this case may have been related to Satoyoshi syndrome. To the best of our knowledge, no prior reports of Satoyoshi syndrome presenting with uveitis or CRVO have been published.

We conclude that uveitis may be complicated by Satoyoshi syndrome. However, more cases are necessary to clarify the relationship between Satoyoshi syndrome and uveitis. Our findings will guide future research and will ultimately help in the development of medical guidelines.

## Author contributions

**Conceptualization:** Toshikatsu Kaburaki.

**Data curation:** Yoshinari Saima, Yoshiaki Tanaka.

**Project administration:** Akihiro Kakehashi, Toshikatsu Kaburaki.

**Supervision:** Akihiro Kakehashi.

**Visualization:** Yoshiaki Tanaka.

**Writing – original draft:** Yoshinari Saima, Toshikatsu Kaburaki.

**Writing – review & editing:** Yoshinari Saima, Yoshiaki Tanaka, Akihiro Kakehashi, Toshikatsu Kaburaki.
